# Mirrored Social Representations of Canadian Caseworkers with Migratory Paths Intervening with Refugees in the Host Country

**DOI:** 10.3390/ijerph18168648

**Published:** 2021-08-16

**Authors:** Lilian Negura, Corinna Buhay, Annamaria Silvana de Rosa

**Affiliations:** 1 Faculty of Social Sciences, School of Social Work, University of Ottawa, Ottawa, ON K1N 6N5, Canada; csimeoni@uottawa.ca; 2 Department of Social and Developmental Psychology, Sapienza University of Rome, 00185 Rome, Italy; annamaria.derosa@uniroma.it

**Keywords:** social representation, refugees, caseworkers, mirrored social representations, social identity

## Abstract

In 2015, the resettlement of 25,000 Syrian refugees in Canada placed a strain on social services. Caseworkers employed in these agencies often come from similar migratory trajectories to those of the refugees. This experiential proximity requires an understanding of the subjective perspectives that caseworkers with migratory paths have of refugees in the context of their professional practice. We analyzed fifteen individual interviews with Canadian caseworkers and conducted field observations of resettlement activities in the Ottawa-Gatineau region using inductive reasoning inspired by grounded theory. Adopting a sociogenetic approach to social representation theory, this qualitative study illustrates how the social representation of refugees among foreign-born caseworkers is highly informed by their migratory past experience, as well as by the social identity and social context from which that representation was socio-generated. Our analysis reveals the *mirror effect* of the caseworkers as a fruitful concept for understanding the identity-otherness dynamics in the encounter between the *distant other* (refugee) and the *self*.

## 1. Introduction

The last decades have seen a significant increase in forced migration [1]. This was a period of exceptionally high numbers of forcibly displaced people in their own and other countries [1]. Following the Syrian government’s crackdown on civil protestors and political crisis in March 2011 [2], more than six million Syrians were displaced internally and externally to camps in the neighbouring countries of Lebanon, Turkey and Jordan. These individuals became “refugees” as defined in the 1951 Geneva Convention under article 33 [3].

In September 2015, the Liberal Party of Canada made the resettlement of Syrian refugees a major component of its election platform, which was extensively covered by the media [4]. The liberal government decided to welcome refugees [5] on the basis of this election promise to rebrand Canada’s humanitarian tradition and re-invigorate its socio-economic potential.

According to a study based on 2016 census data [6], more than 25,000 Syrian refugees were admitted to Canada between November 2015 and February 2016. Furthermore, Canada went on to welcome 46,700 refugees on humanitarian grounds in 2016 alone, setting a new record since the immigration act was adopted in 1978 [7,8]. This special mission, referred to as “Operation Syrian Refugees”, was Canada’s response to the humanitarian crisis in Syria. Over the course of one hundred days starting in November 2015, private sponsors, non-governmental organizations, as well as provincial, territorial and municipal governments and international partners, worked closely to resettle the Syrian refugees in Canada [5]. Canada’s major political motivation for the resettlement of Syrian refugees was, according to J. Garcea and K. Daniel, a strategy to improve its reputation on a global scale as a generous and welcoming country [4].

The mission around refugees also served an identity function. The generosity and goodwill towards Syrian refugees were depicted in the media as the reflection of Canadians’ “true” identity [9]. However, V. Tyyskä et al. [10] contend that while the media emphasized the generosity of Canadians, at the same time they continue to depict refugees using a misleading bipolarized representation ranging from being needy to being a possible threat.

Despite these problems, the media attention has helped to mobilize citizens to participate in the integration of Syrian refugees. The willingness of Canadians to help refugees is explained by some researchers [11] as resulting from empathy towards the refugees, who have experienced severe physical and mental challenges related to war trauma and to radical changes in their socioeconomic conditions [12,13]. Nevertheless, other studies that analyzed the resettlement experience of Syrian refugees [14,15,16] highlighted the refugees’ potential resilience, which enables them to adapt to the host society [17].

Existing studies emphasize the inadequacy of resources allocated by Canada to meet the needs of refugees, such as language and skills training, social networks, as well as employment [18] and highlight that these needs are generally greater among refugees than among economic immigrants [19]. It is no wonder, then, that helping professionals feel overloaded and poorly equipped to adequately address the needs of refugees [20].

In addition, these caseworkers have also been feeling increasingly overwhelmed by the urgent, unprecedented, intensive and ongoing demands placed on them. As front-line professionals, caseworkers, through their ongoing social and professional interactions with refugees, have experienced a gradual erosion in their own empathy, which may have changed their perspective on refugees. In fact, it appears that working closely with traumatized populations such as refugees might revive old emotional wounds, and this work draws a great deal upon individual resilience [21]. This is even more true when caseworkers have little or no previous experience in social work or trauma management [22].

Research has revealed the importance of cultural-competence assets and sensibilities in working with refugees [20]. In this context, M. Fennig considers the cultural adaptation of mental health interventions as a promising and pragmatic approach to service delivery [23]. To help address the cultural needs of refugees, the majority of caseworkers recruited in 2015–2016 to work with Syrian refugees were themselves former migrants or refugees [24]. In terms of social or cultural background, these workers also tend to identify with refugees [25]. The authors report that resettlement organizations often hire them because of their cultural knowledge or linguistic skills, which are mobilized to better understand the experience of refugees and intervene with them. It is therefore important to explore the subjective perspectives of these caseworkers that are rooted in their personal history, their social identity and their assigned role as community representatives.

Therefore, we investigated first how the caseworkers’ social representation of refugees is anchored in their own migration pathways and second how this process affects their professional experience. Social representation is an expression of lay knowledge, a theory of common sense about an object of significance to a social group [26,27,28], produced by natural logic [29] and social thought [30] and orienting individuals in their social and professional environments [31]. That concept enables “communication among members of a community through a shared code for social exchange and for naming and classifying various aspects of the social world including their individual and group history” [32].

Social representations are created and transformed through two processes: anchoring and objectification [28]. While, through objectification, an abstract concept such as “refugee” becomes concrete and operational in everyday exchanges, then, through anchoring, this object becomes familiar. This happens through the insertion of the object within a known system of reference, such as lived experience [33], in a manner that facilitates the mastering of the representational object—namely, refugees.

Social representations also have an identity function [34]. Hanson-Easey and Moloney [35] found that in the social representation of refugees among students in Australia, the refugees’ place of origin has an important role in associating refugees with certain socio-political contexts. For Canadian caseworkers with an immigration background trajectory, however, the places of origin of refugees may have a different meaning, given the likely anchoring of their social representation of refugees in their own personal lived experience.

Canadians’ involvement in refugee settlement could thus be understood as identity work. According to some studies [9,36], the process of refugee hosting and sponsorship policy involves the transformation of refugees into New-Canadian citizens. In this way, Canadians who identify the “other” with “us” also actively enact their own identity as Canadian citizens. Analyzing the links between caseworkers’ social representation of refugees and their own identity and how those links contribute to specific social-intervention experiences (pp. 11–40 in [31]) with refugees is at the heart of this study. Specifically, we questioned how the social representations of refugees among Canadian caseworkers with a migratory path or New-Canadian caseworkers (“New Canadian” is an expression that in Canada means a Canadian of immigrant origin. This meaning can be found in the Collins dictionary: https://www.collinsdictionary.com/dictionary/english/new-canadian, accessed on 13 August 2021) have emerged during the 2015–2017 Syrian resettlement and shaped their identities and their lived experiences of this relocation process. The next section deals with our methodological approach and research strategy, which are anchored in the constructivist epistemological stance.

## 2. Method

### 2.1. Research Design

To collect our data, we adopted a multi-method research design [37,38] that integrated two methods: semi-structured individual interviews and field participant observations (Table 1). This pluri-methodology is inspired by grounded theory [39] and allows for the triangulation of methods. As our method uses inductive reasoning, our analysis of the data was done at the same time as the collection: we immersed ourselves in the details, the specifics and the context of the data to discover themes and construct broader ideas. Thus, the analytical categories emerged from the data without presupposing anything a priori; instead, we engaged in a posteriori construction of the categories.

**Table 1:** Multi-method research design.

Method	Participants and Resettlement Activities	Location and Time Frame
Semi-structured interviews	15 caseworkers, having at least 6 months of professional experience with the refugee population and being currently involved in the resettlement of Syrian refugees (For details see Table 2).	Ottawa region, November 2016–November 2017
Participant observation	7 social and cultural activities for the Syrian refugees (For details see Table 3).	Ottawa and Gatineau regions, January–November 2017

**Table 2:** Characteristics of the research participants.

#	Fictitious Name	Country of Origin	Formal Migration Status
1	Lena	Algeria	Economic migrant
2	Kalil	Iraq-Kurdistan	Refugee status
3	Latifa	Palestine	Economic migrant
4	Luis	Venezuela	Economic migrant
5	Boris	Bosnia	Refugee status
6	Mouna	United Arab Emirates	Economic migrant
7	Melissa	Australia	Tourist who later decided to stay in Canada
8	Youssef	Lebanon	Economic migrant
9	Maria	El Salvador	Economic migrant
10	Sara	Morocco	Economic migrant
11	Ruth	Israel	Economic migrant
12	Hatem	Iraq	Refugee status
13	Maziar	Iran	Family reunification
14	Nora	Jordan	Economic migrant
15	Sergio	Peru	Economic migrant

**Table 3:** Characteristics of observed social and cultural activities.

#	Activity	Location	Organizer	Time Frame
1	Greetings at the Airport	Ottawa International Airport	Local United Nations	January–February 2017
2	Initiation to Embroidery	Church basement, Ottawa	Local resettlement organization	January 2017
3	Zen Session	Church basement, Ottawa	Local resettlement organization	January 2017
4	Garden on the Balcony	Church basement, Ottawa	Local resettlement organization	March 2017
5	Happy Birthday Canada	Church basement, Ottawa	Chinese Social Services Organization	March 2017
6	Friends of the Family	Local resettlement organization Ottawa	OCISO	February 2017–April 2017
7	Farewell dinner	Local Christian church Ottawa	City Hall Ottawa—Community College and Resettlement agency	June 2017

### 2.2. Participants

We used a standard non-representative purposive sampling of 15 caseworkers. The research participants were recruited from the resettlement agencies that accompanied the Syrian refugees in Ottawa. The inclusion criteria for purposive sampling comprised the following: being a caseworker, having at least 6 months of professional experience with the refugee population and being currently involved in the resettlement of Syrian refugees (see Table 2). Because our broader goal was initially to study the social representation of refugees by caseworkers involved in the resettlement of Syrian refugees in Canada, we did not set being a former migrant or refugee as an inclusion criterion. This characteristic of our participants emerged from the field when we observed that almost all of the caseworkers had a migration background. Due to the fact that our methodology was inspired by grounded theory, we used the principle of theoretical sampling to continue recruiting participants. Theoretical sampling is defined by Glaser and Strauss [40] as “the process of data collection for generating theory whereby the analyst jointly collects, codes and analyses his data and decides what data to collect next and where to find them, in order to develop theory as it emerges” (p. 42). In this way, the participants were selected based on data provided by previous participants (questions emerging from the data) and the ability of each participant to contribute to a better understanding of the research object through their own knowledge and experience.

Three of the caseworkers recruited were refugees (two from Iraq and one from Bosnia), most of them had a background that was more in line with the category of economic immigrants, and one had chosen Canada as host country after a touristic experience. However, all of them had at least 6 months of previous experience with migrants and refugees coming from war zones (Iraq, Afghanistan, former Yugoslavia, etc.). We therefore did not seek to identify only participants with a refugee background. Our position is that the reality of people moving to wealthy Western countries, whether under the label of refugees or economic migrants, is deeply complex and specific to each individual.

We are not alone in this critical stance towards these opposing binary categories [41] (p. 50). Other prominent authors in the field have argued in this vein. These categories oversimplify a complex migration process by ignoring the fact that often people with very different motivations end up together on the same journey and that they can change status at different points in their journey and can fit into several categories at the same time [42]. Koser and Martin [43] mentioned that these categories do not consider the evolution and diversification of migration profiles and situations in recent years. Moreover, these categories refer to countries of origin and destination, to “objective” or “declared” causes of displacement or to the duration of displacement and do not take into consideration the actual experience of these people [41]. de Rosa et al. [44], through the analysis of 611 documents related to the institutional and policies-driven discourse within different geo-political governmental scenarios, have detected a plethora of definitions of what it means to be an immigrant. The long list of labels that the authors have examined shows the relevance of the “(de)personification” and “(un)naming” of the (im)migrants as an out-group, offering a new lecture key for the “objectification” and “anchoring” processes that build social representations. When we constructed our sample, we wanted to capture the diversity and complexity of the migration experiences of caseworkers.

### 2.3. Data Collection

Fifteen semi-directed interviews, conducted in English or French with caseworkers involved in the resettlement of Syrian refugees in Ottawa and Gatineau region in Canada, lasted between 30 and 150 min. Since the participants and all the researchers in this study were fluent in Canada’s two official languages, no translation was required. The interviews were audio-recorded or transcribed by hand if participants requested to not be recorded. The discussions were organized around the following themes: the ideas or associations caseworkers make when they think about refugees, their relationships with the refugees, the personal and professional trajectories of migrant caseworkers, their professional training related or not related to social work, their professional work with the refugees’ target group, their professional practices with the refugees around resettlement, their views and perceptions on being both a migrant and a caseworker and the impact of that dual identity on their work. This process aimed to capture the social representation of refugees among Canadian caseworkers with a migration path.

The semi-structured interviews allowed us to achieve the first objective of our study, which was to understand how the social representation of refugees is anchored in their own migration pathway. To better understand the role of this social representation in their professional experience, which was our second objective, we implemented participant observation of the activities carried out by the caseworkers related to the integration of Syrian refugees.

The triangulation of data from the two data collection methods, semi-structured interviews and participant observation, aimed to give to our qualitative approach not only better validity but also, and more importantly, rigor, breadth and depth [45,46] in studying the social representations as an essential component in the construction of the social reality [47] of caseworkers. Finally, participant observation is a key method in the sociogenetic approach to social-representation research, which our study falls under because of its ability to capture the social representations as a historically, culturally and socially situated process [33]. The last two points allowed us, as explained latter in this article, to ensure the trustworthiness of the entire research [48].

The second author participated in seven integration activities, whose objective was to guide and orient refugees into the host country, Canada. Moreover, the purpose of the activities was to help Syrian refugees navigate into the social system in order to better understand it and use it on their own.

First, the managers of the Ottawa-Gatineau resettlement agencies were contacted and asked about the services and programs for Syrian refugees. In fall 2015, right after the liberal government was elected at federal election in Canada, agencies received a certain amount of budget to implement specific social and cultural activities for the Syrian refugees (e.g., language classes, welcoming activities at the airport, etc.). The caseworkers interviewed were also in charge of the organization of these activities, so we had the opportunity to be invited to give a better understanding of the current resettlement and to see at first glance and first-hand the current situation of the resettlement.

Consequently, the second author participated in and observed most of these activities by helping the set-up and the organization of them as a volunteer. The activities were held between January 2017 and July 2017. During that six-month field investigation, the interactions between Syrian refugees and the Canadian caseworkers were systematically observed. Participant observations were conducted in the form of full observations. All the observations were written and noted in a running observation record according to themes (type of event or activity, social and cultural norms, roles of organizers and caseworkers, etc.).

One of the first activities was *Greetings at the Airport.* The welcoming at the airport of Syrian families by a representative of an international organization and its host group was certainly the most relevant event of this observation work. The “function” attributed to the researcher was to distribute the symbolic and representative objects of Canada: a miniaturized version of the Canadian flag; a polar-bear-shaped teddy bear with the maple leaf; and a welcome kit, a kind of toolbox containing the map of the area, local treats, stickers for children and a list of addresses from social organizations that can help newcomers in their acculturation process.

A second activity, *Garden on the Balcony,* took place in order to teach the refugees how to garden and make plant cuttings (mint, coriander, basil and cherry tomatoes) in pots. These former farmers now live in apartments whose balconies may accommodate small pots.

The next activity was the making of a patchwork, or *Introduction to Embroidery.* Refugees made embroidery in the shape of a maple leaf, an activity representative of the Canadian flag. This activity graciously invited women and their children to make embroideries to give them a taste of their new welcoming land.

Another activity called *Zen Session* gave Syrian women makeup or hairdressing tips and offered them other ways to de-stress after difficult times. These were fun activities to decompress from the week, in which the intense six hours per day language sessions create much stress.

In addition, the involvement of the refugees in the *Friends of the Family* program showed the link in the chain of solidarity that was being forged between the different communities in Canada. The coaching was flexible, offering one’s time and friendship, the guiding principle being becoming friends with a newcomer and sharing with them what we think we know about Canada. Being a friendly support for a Syrian family in order to alleviate their loneliness in their new environment was a successful program for both Syrians refugees and “friends”.

*The Happy Birthday Canada cultural evening* was an activity that was dedicated to celebrating the 150th anniversary of Canada and its newcomers. Again, our role was to distribute Syrian dishes prepared by a former Damascus chef to a hundred guests and make sure that everything went according to plan. Music, traditional songs and YouTube videos evoking the images of the past all helped to transport these families back into the time “before Canada”.

Finally, 2017, with its wide variety of integrative activities, ended with a *farewell dinner and the presentation of diplomas of participation to volunteers*, rewarding the sustained efforts of many volunteers representing Canada as both multicultural and humanitarian, devoted to the management of “crises” and to the welcoming of refugees.

### 2.4. Data Analysis

A content analysis was initiated to synthesize and organize the qualitative data generated by both the semi-structured interviews and the participatory observations. According to Negura [49], the object of content analysis is the communication that allows the formation of social representations (p. 2), which makes it particularly relevant to our study. In an inductive approach linked to grounded theorizing, the analysis occurred in a constant back-and-forth with the data collection.

First, once all the interviews had been transcribed, they were inserted into NVIVO10 software, where the field notes were processed and analyzed manually. The initial reading of the material allowed the capture of the main points that emerged from the data.

Next, a systematic reading of the interviews and field notes revealed common themes observed or discussed by participants that frequently reappeared in the analyzed texts. The textual segments were coded into “units of meaning” [50]. Following the creation of categories called “nodes” in the software, we clarified the meaning of these elements of discourse and highlighted connections to the research question. This coding, when completed, suggested a categorical classification for understanding the formation of the social representation of refugees among caseworkers.

Social representations require a particularly close analysis. In line with the sociogenetic perspective [33], we sought to understand how the social representations of the refugees among the caseworkers emerged from their personal histories, particularly their migratory histories, and how these representations were reflected in their professional practices with refugees.

In order to meet the highest quality requirements for our qualitative research, we followed the recommendations made by the Task Force on Resources for the Publication of Qualitative Research of the Society for Qualitative Inquiry in Psychology, a section of Division 5 of the American Psychological Association [48]. This task force proposed the concept of methodological integrity as the basis for trustworthiness in qualitative research and suggested that it be assessed through two composite processes: (a) fidelity to the subject matter and (b) usefulness in achieving the research objectives. The fidelity to the subject matter is “the process by which researchers develop and maintain allegiance to the phenomenon under study as it is conceived within their tradition of inquiry” [48] (p. 2). The utility in achieving research goals is “the process by which researchers select procedures to generate insightful findings that usefully answer their research questions” [48] (p. 2).

In order to meet the criterion of fidelity to the subject matter, we chose, as recommended by this task force, to cross-reference data from two distinct methods: semi-structured interviews and participant observation, both of which improve the fidelity of the research, as these methods provide data that can “shed light upon variations in the phenomenon” [48] (p. 11). To meet this fidelity criterion, we also chose to have in the sample not only caseworkers with a refugee background (three participants) but also one participant with an atypical migration background (from Australia) and participants with a different migration experience (economic migrants). This allowed us to put into perspective the different migration backgrounds of the caseworkers and the role of these backgrounds in their social representation of refugees.

Two of the researchers have migration experience, and one of the researchers has experience working with Syrian refugees in Canada and has been a social worker for the past 30 years. Two of the researchers have experience in the migration research field and have published in this area. This diversity of experience of the authors of this article in migration and intervention with refugees allowed for a good management of the researchers’ perspectives in the analysis of the data in order to improve the fidelity of the analysis, as recommended by the task force.

To meet the second criterion, that of utility, we avoided identifying decontextualized procedures but rather situated our data in the context of the Syrian refugee crisis and took into account the political context of the federal election in Canada in the development of the research instruments. In order to achieve the research objectives, which were to capture the impact of the lived experience of migration of the caseworkers on their representation of refugees, we chose data collection methods that were not very restrictive for the participants and that allowed us to have access not only to the caseworkers’ discourse (semi-structured interview) but also to the manifestations of the representations in their behaviours (participant observation). This maximized the utility of the research, as recommended by the Task Force [48].

## 3. Results

### 3.1. The Dual Representation of Refugees

Participants felt overwhelmed and had difficulty finding the right words to describe refugees. Multiple articulated and only apparently contradictory meanings came to mind when caseworkers were asked to provide words that they associated with refugees. According to our caseworker research participants, the refugees needed help, support and assistance due to their lived traumatic experiences. Wars and political terror have in fact left lifelong marks on the Syrian refugees’ bodies and minds. They therefore needed help from the host society because they were vulnerable: “words to describe refugees are ‘vulnerability’ (because they get government assistance), ‘traumatic past’” (Hatem) (All fragments with quotation marks in the Section 3 are quotes from participants.). “Their experiences traumatized them; I see that in their faces; they show clear signs of trauma and anxiety. I can see the signs, read their faces. They look lost, traumatized; they are always looking for information and directions to go to some places, to accomplish things basic to us but not them. They look confused, as well as overwhelmed and anxious. Their thoughts are always interrupted. They lack focus and concentration” (Latifa).

However, participants also expressed more empowered images of refugees: “For me, a refugee is someone who is resilient, who may not have chosen the path they have been on, but hopefully they can find their way in it; because they are safe now, everything will fall into place” (Mouna). The fact that they have had an extremely difficult journey, full of danger to their lives and the lives of their loved ones, demonstrated, according to participants, that refugees are ultimately resilient, that they have an immense capacity to cope with difficulties and therefore an exceptional potential to adapt to the host society: “We tend to see migrants as always lacking skills. However, every day I witness their strength and competencies” (Ruth). Moreover, the lives of refugees showcased examples of humility and humanity; they provided rich stories that should be heard and that can serve as life lessons for other Canadians and immigrants: “When I hear the word ‘refugee’, what comes to my mind is resilience, a group of people who went through hell and came out alive; refugees have a lot of potential because by having gone through hell, they teach us a lesson of life, of humility; we need to be interested in their stories and in the daily lessons that they can teach us; we need to validate their words; through validation, you can better understand their situation” (Latifa).

Sometimes, the personal experiences of the caseworkers were used to further support this positive view of refugees as resilient and strong people: “I went through the same difficulties. Going through it was not that easy. I know the challenges of being from abroad. It is very tough to be an immigrant” (Kalil). This description projects optimism that refugees will be able to find their place in their new society: “I am a Kurd, a refugee myself. I love English, literature and the classics, music and books; these are my passions. I came as a refugee to Canada, and in one week, my first week, I wanted to work but couldn’t, so I volunteered in this resettlement agency in Toronto, you know my sixth day in Canada I worked as a translator. Canada is good to me. So I wanted to give back to others” (Kalil). Through their own life trajectories, caseworkers demonstrated the potential for success and could work with empathy, experience and knowledge to offer help to refugees: “Being an immigrant myself from Lebanon, it is an asset to be an immigrant and serve other immigrants; it is a huge asset in terms of understanding cultural nuances without the need to ask questions. My immigrant identity creates a sense of familiarity, a shared place of origin with similar sub-cultural values; there is another advantage to being an immigrant; my immigrant identity helps to put the client at ease: it helps them to feel free to describe, to express their experience without judgment, without the feeling of having to put a mask on many things; you have to put your true self out there” (Youssef).

The participants’ discourse revealed a dual representation of refugees as both “strong” and “suffering”: a few words “are not enough to describe a refugee. I have many words in my mind to say about refugees. So, I think that ‘refugee’ is like: ‘suffering’, ‘strong’, ‘they left their country’, because of the war they are ‘strong’, ‘human’, ‘fragile’, also ‘newcomer.’ They come from war, but they are ‘strong’” (Hatem). Vulnerable but also resilient; they have lived through “hell” but have emerged “strong” from this ordeal. Additionally, they have qualities that allow them to succeed in their new life. Refugees are also, according to participants, aware of their human rights to be free, dignified and to live with safety, rights that they want to defend and that were the reason they chose this country, their new home: “A person who is seeking asylum, they want a place they can call home, somewhere where they feel that they have their rights and dignity, somewhere where they know they are safe, a safe place. They do not have to look over their shoulders; they are free human beings with human rights” (Latifa).

To conclude this section, the caseworkers with migration paths who accompanied new migrants in Canada had a dual social representation of refugees as being both vulnerable and resilient. This representation was anchored in their own migratory experience, a process that we will detail in the next paragraphs.

It should be noted that these opposing and only apparently contradictory images of the refugees by the caseworkers do not reflect the polarized views of immigrants widely circulating in society and media [51,52]—namely, their depiction as “victims” or “invaders” corresponding to politicized views of the “other” as a “useful resource” or as a “threat” to the host country. In the case of the caseworkers interviewed in this study, the different anchoring of the representations was not driven by exclusion processes of “othering,” but by the empathy-informed wish to recognize in the refugees the vulnerability of forcibly displaced people or the resilience and strength to face tragic human conditions and overcome difficulties with an exceptional capacity of adaptation.

### 3.2. Mirror Effect or the Process of Anchoring of the Social Representation of Refugees

#### 3.2.1. “Going through the Same” Experience

“Going through it” implied, for our respondents, to grieve the traumatic past. However, nostalgia dominated the discourse: “I left my country a long time ago … yes, I remember, sometimes it seems like it was like yesterday … (o.t.)” (Boris) (All quotations in French translated by the authors into English are indicated by o.t. (our translation)). The metaphor of the “mirror” is useful here because it symbolizes the constant interaction between the outside world and the construction of the self. Most of the participants self-identified as first-generation migrants, pioneers for their families. The mirror, then, kept reflecting the images from the past: “working with refugees, there are constant references to one’s own refugee or immigrant background (o.t.)” (Maziar).

It seems that the migratory trajectory involves suffering, sacrifice and “learn[ing] to manage your emotions (o.t.)” (Sara). It also entails thinking and hoping that “what worked for me can work for others (o.t.)” (Lena). Caseworkers helped refugees to “understand the host society, otherwise you will suffer” (Maziar), and encouraged them to feel relieved that “their families have arrived safely” (field notes).

The close professional interaction with refugees could trigger in caseworkers memories of their own experience and generated stress and exhaustion. Thus, the first step towards the anchoring process of social representation of refugees was to “go through the same” experience, which enabled caseworkers to capture the complexity of being a migrant, with all the dimensions of that experience. The second step described the social process of the accompanying of refugees following the adoption of a new social identity, the identity of a caseworker, and how this duality of identities played a role in the caseworkers’ own integration in the host country. Having gone through the same migratory process, caseworkers were more likely to understand the emotions and the challenges of the integration into a host country. Therefore, two compatible identities emerged, arising from two specific contexts and lived experience which enriched the professional resettlement work.

#### 3.2.2. Professionally Supporting the Refugees

Since first-generation migrant caseworkers went through some aspects of the migration route, which triggered their own past experiences, they experienced continuous mirroring of the first stages of their own migration. Although social support remained at the heart of the caseworkers’ professional interventions, most of them did not have any previous experience in social work, especially with traumatized populations (e.g., PTSD).

The linguistic skills and cultural competencies of caseworkers therefore remained the main criterion for their hiring: “I speak the same language, and I understand the culture” (Latifa). As a result, the mobilization of cultural competencies, including linguistic, cultural, social and geographical assets, as well as knowledge about migration, was viewed by Canadian migration authorities as essential to working with refugees. Mostly, however, it was the ability to participate in the cultural networks and build bridges between the newcomers and the locally established community that was valued in caseworkers with migration paths.

An example of the mobilisation of the migratory experience of caseworkers with migration paths was during the *Canada 150* activity, which took place in the presence of public officials and community representatives. By translating and serving as mediating bridges between the refugees and other Canadians, the caseworkers and volunteers contributed to narrowing the cultural gap between refugees and Canadians and fostering a sense of home for the newcomers. Sometimes what might seem like an ordinary activity may appear very significant to a newcomer and become a moment of solidarity and newfound friendship: “they feel like home; they were back home, again” (field notes); “learning a foreign language is hard, they are here today; that’s good. They just enjoy and relax, don’t need to think too much about problems” (field notes). Integration activities could be experienced as opportunities to come together and collectively share laughter, frustration, and learning experiences, “spending time together, like back home” (field notes).

Furthermore, the cultural and professional proximity experienced by the caseworkers perpetuated the “mirror effect” and harboured ambiguous feelings among them. As they worked to ensure the implementation of the standards imposed by the institutional mandate, caseworkers might have felt at odds with their loyalty towards their service users. They were torn between the fear of confronting their past and the needs involved in the refugees’ acculturation in the host society. For instance, a caseworker might expect the newcomer “to fit-in quickly:” “They are like children who must learn to run, to read, to speak. The refugee must learn to live in Canada. Just as we did (o.t.)” (Sara).

Integration may be experienced as somewhat “forced:” “we have been pushed to force them to integrate; we have no choice, either… too fast, everything is too fast… you must learn to put your distress aside” (Melissa). Integration is a process, and it emerges from a need to guide and orient newcomers into the host country by implementing programs and services to ease the socio-economic and cultural blending between many cultures.

Sometimes perceived as agents of accountability or normativity or as “moral entrepreneurs” [53] who establish and enforce the rules, the caseworkers expected newcomers to make the same effort as they had done: “follow the law, as we have done” (Maria). This might lead to imposing Canada on refugees as their new country by minimizing their past: “fresh off the boat and they already need to speak English as quickly as possible, get a job, a house, a new life! We ask a lot from them!” (Latifa). This identity imbalance required rigorous adaptation strategies to avoid favoring one identity over another. Being in between worlds called for accepting the uniqueness of the caseworkers’ own plural identities, which impacts social support practices and interactions. “I was an immigrant, me too” (Boris) is a reminder of how caseworkers confronted their own past in the process of helping refugees. Knowing the situation from inside made caseworkers better qualified to work with migrant populations, allowing them to juggle the distance (as caseworker) and proximity (as former migrants). Whatever the uniqueness or similarity of the experience, an empathetic approach made it possible to better identify the vulnerability of the “other.” Accompanying newcomers also involved referring them to other services or organizations better suited to meet their needs. Indeed, the interviews revealed the fragmentation of services and the lack of coordination that undermines social interventions. This disorganization added to the stress of both the service users and the caseworkers: “quickly once you arrive here, you have to go through all the administrative procedures, you have been forced to integrate, you have no choice … (o.t.)” (Boris).

Therefore, providing quality community services to these newcomers required a sound knowledge of the factors that affected their mental health [54,55,56,57]: “The added value is the human value that we add to make people feel welcome, comfortable and to belong like any human being” (Sergio). By expecting immediate accountability and effective integration from newcomers, the host country should, according to our respondents, take responsibility for establishing adequate structures. A caseworker emphasized that responsibility: “They called me and yelled at me because they thought we didn’t do enough for the refugees! They saw children playing with sandals in the streets and couldn’t understand why we didn’t give them warm shoes and clothes! For them, it was unacceptable! But we took care of them!” (Maria).

However, another caseworker questioned the relevance of programs and integration activities: “lots of agencies seem to work on their own in silo; there is no coordination because it’s in silo work, which not only exhausts the stakeholders, but this bombardment of information can be disturbing to them (o.t.)” (Sara).

Because caseworkers have to respond to traumatic and challenging situations experienced by refugees, their psychological well-being could be affected, according to their testimony, particularly if they never had the opportunity to work with similar situations: “We were all swamped, fed up, burnt-out and very tired. It was challenging” (Ruth). The “mirror effect” continued and may thus have had a double-edged influence on both the caseworker and the refugees.

#### 3.2.3. Stress: The Dark Side of Empathy

Another important dimension of this journey was the compassion fatigue, weariness and stress felt by the caseworkers who “have given much” (Sara) during the phase of resettlement and assistance. When it comes to describing compassion fatigue, authors use different terminologies. Freundenberger [58] speaks of “burning inside”, pressures created in the domestic sphere due to the devotion to a cause but also due to the concern and empathy extended to another human being. This fatigue can be understood as a result of the repeated confrontation with the distress of others, the inability to respond adequately to demands for relief and the inevitable and repeated failures in the care of suffering patients [59]. The confrontation with the distress of others shapes caseworkers’ representation of refugees. Indeed, it is perhaps at this stage that the “mirror effect” captures the ambivalence of the multiple identities formed during the migration route.

The main problems encountered in the social support of refugees were often characterized by the trauma of exile or the circumstances of forced migration. In addition, the professional role of caseworkers was limited to assisting refugees in their practical lives (housing, transportation, access to medical and social care, education for children, language courses, job search, etc.). When it came to psychological or emotional situations, caseworkers did not have the required training, the staff or other resources to provide such interventions. Most often, they referred refugees to specialized services (clinics, psychologists, psychiatrists, etc.). There was a gap between what was needed and what was offered, largely due to budget constraints, which forced caseworkers to review the objectives and priorities of what could be offered to newcomers.

The testimonials of caseworkers reflected elements of emotional fatigue: “You have to manage that too. I learned to manage that with time; the first few times, it was very difficult for me; I cried all the time with everyone who is sharing a similar story as mine and now I’m able to manage the emotions (o.t.)” (Sara). Witnessing the pain or listening to the stories of the lives of refugees, burdened with traumas and tragedies, resonated with community services professionals, who themselves had often pursued a migration route, although not necessarily from the same geo-cultural area or under the same conditions as their service users. Caseworkers knew that the refugees’ journey continues in the host country: “Even though it is sometimes painful, we feel, accept, taste and endure suffering and do not erase it from our mind. It is here and not there. One day it will be fully understood, not now. It is a matter of time” (Nora).

The main organizer of the Canadian Flag Patchwork activity, in which Syrian women and their children were invited to make embroideries for the 150th anniversary of Canada, attentively organized the event, with the assumption that showing the participants a generous outpouring of compassion and giving them warmth and attention would create a sense of community and help to ease the pain of the past: “Me, when I arrived here, we didn’t have that. No activities” (organizer, field notes). We can thus only imagine the feelings of indebtedness and perhaps embarrassment from the beneficiaries of these activities who did not have a choice other than feeling grateful to the organizers.

Furthermore, an asymmetrical relationship was observed during field activities in which some newcomers felt compelled to participate and obliged to give back to society. The demand to give back to society presupposes that newcomers would adapt quickly and effectively to new ways of thinking and doing in the host society: “We quickly taught life skills so they could understand Canadian values and learn to live here” (Melissa).

In conclusion of this section, the “mirror effect” was an experience felt by our caseworkers because of their dual identity as former migrants and as helping professionals. Caseworkers were forced to align their working methods with the institutional requirements of the community centers in which they worked. With their cultural affiliation and their institutional loyalty often at odds, caseworkers appeared to navigate into troubled waters and tried to continue their work out of concern for the wellbeing of the users of their social work.

## 4. Discussion

The social representation of refugees expressed by our participants incorporates two realities that at first glance seem to be contradictory. For our participants, the caseworkers who themselves have a migration background, refugees are represented as both vulnerable and resilient.

### 4.1. Refugees and Vulnerability

In Latin, vulnus (wound) or vulnerare (to wound), to be a vulnerable, implies a fragile person “who can be wounded and easily harmed at the core” [60]. Certain social groups (e.g., migrants, women) are labeled as such because the stigma of vulnerability is overused and perpetuated outside of the context in which the individual evolves and survives. Indeed, what makes a refugee vulnerable is their precarious situation as an internally or externally displaced person [61]. Being in a socio-cultural environment where all communication codes have changed meaning inevitably and naturally leads to temporary moments of vulnerability [62]. Often not in control of daily situations, refugees depend on international organizations and community centers that have developed specialized and highly professionalized expertise to assist them [63].

Nonetheless, certain international structural barriers, in addition to those imposed by the host society, threaten refugees’ social identity [64]. Refugee organizations and service providers become targets and instruments for implementing the socio-political imperatives of international actors [65]. Refugees often claim autonomy [66] from these international humanitarian and social organizations that unilaterally manage refugee migration paths. At the same time, these organizations perpetuate through their practices the image of refugees as vulnerable individuals.

The social representation of vulnerable refugees is therefore not only a reflection of a precarious situation during the resettlement process; it is also an image that is maintained by institutional requirements and reproduced by social actors as an othering effect [67]. Our data show that this social representation of refugees as vulnerable is incomplete, according to the caseworkers with an immigration background. Furthermore, this representation may stigmatize refugees, dispossess them of their expertise and subject them to the unwanted intervention and burden of expert social services and humanitarian organizations.

This social representation of the vulnerable refugee supports the idea that the compassionate host society does not offer its welcome freely and disinterestedly. In exchange and in return for help, the recipient is expected to give something back to the generous giver. Benefiting from a wide range of social services designed to facilitate their integration, recipients are expected to express their gratitude [68,69]. Demanding gratitude from vulnerable individuals means that if they hope to gain social support and charity from the host society, refugees must follow the norms of the host society and live as members of that society. These expectations symbolize the form of adaptation and integration required of a newcomer [70]. The extensive literature on migration and integration has often focused on the conflict between home and host cultures [71]. Refugees are therefore invited to better understand and respond to the expectations and norms of the host society [72].

### 4.2. The Mirror Effect

Reflecting the diversity and multiculturalism of the host society, refugee vulnerability continues to be a hegemonic representation [27] that persists over time and is deeply embedded in the collective imagination [73]. However, vulnerability is also a subjective experience that is expressed in the body’s history and results in emotional trauma [74,75]. Caseworkers with similar personal trajectories to the recipients of their service therefore often face a dilemma between showing compassion and fostering integration. This is a strong tension they feel in their professional relationship with refugees. Caught between two worlds, often recruited to professionally support their social work users on the basis of cultural affinity, this position prompts them to use their personal knowledge from both their past and present. This dual social positioning [76] places caseworkers at odds, as if they are in front of a mirror in which the other is reflected. By perceiving others as mirrors of themselves, these individuals become intentional agents [77].

We noticed among our participants a tension generated by the power relationship between them as caseworkers, representatives of the host society, and the refugees, newcomers in need of help. This social positioning is supported by the hegemonic social representation of refugees as vulnerable people. According to Gillespie and Cornish [78], the voice of some in our highly differentiated society is more influential than that of others in legitimising dominant social representations. Replicated in the intersubjective sphere [79], this representation legitimizes the power relationship between caseworkers and refugees.

As our data have shown, it is not only the social position as representatives of the host society that intervenes in the relationship of caseworkers with refugees. The lived experience of caseworkers who have had a migration background also plays a role. This reflection of the refugee-service users’ own experience generates a tension that we have called the mirror effect. Indeed, social representation is not shaped only by the social position we occupy but also by our life experiences, which mark through our emotional reception of life events our constitution as unique individuals. The resonance of the self in the other thus diminishes the asymmetrical dimension of the relationship with refugees and consequently affects the representational content. According to G. Breakwell [80], the bi-univocal relations between identity and social representations are driven by intragroup and intergroup dynamics. As the process of self-reflection, where individuals and groups look in the mirror, can be both instructive and transformative [81], the mirror effect allows for a self-awareness that leads to deontological decisions that deny possible wrongdoing in relation to the other [82].

### 4.3. A Dual Representation

The trajectories of the caseworkers trace the plurality of the self that tends towards both strength and vulnerability. Their social representation of refugees is thus dual. Both resilient and vulnerable, refugees actively construct their destiny while being shaped by powerful global forces beyond their control [63]. The contemporary globalized world reflects this duality and diversity, where multidimensional identities emerge and facilitate navigation between multiple contexts. When individuals are affected by the consequences of international mobility, they are faced with multiple and complex realities that lead to migratory identities [1,83].

Bouquet and Jaeger pointed out that refugee-integration professionals, like any other professional working with disadvantaged people, are confronted with permanent contradictions between public order, professional ethics and the need to take into account the daily reality of migrants’ journeys [23]. These professionals are also challenged in their relationships with refugees by institutional norms and social policies over which they have little control [84].

On the one hand, their membership in the group of caseworkers whose mission is to work on the social integration of refugees obliges them to classify refugees into the categories provided by “social boxes” because, in order to benefit from temporary social support, the individual must “fit into the box” [85]. On the other hand, their personal history teaches them that this box does not correspond to the often very complex situation in which the refugees find themselves. The caseworkers, listeners to the refugees’ life stories, experience a dilemma. They have to choose between “a normalizing function and a contribution to social change” [85], with this change being a mission of public (or political) interest [23] but also in the interest of the refugees. The complexity of refugee identity is that it integrates in an intersecting way all the identities borrowed and absorbed during the refugees’ life trajectory. For our participants, the constant labelling of refugees as vulnerable and governed by emotions such as fear and suspicion, prevented them from showing other facets of their identity, such as adaptive strength, resilience, resistance and creativity.

To conclude, the experiential dimension as a self-reflection process through the *mirror effect* allows the caseworkers with migration backgrounds to give voice to their “empathy” towards refugees, who are perceived at the same time as vulnerable, fragile victims and as strong, resilient individuals. This intertwined dual representation reflects the shared complexity of the life experience of both refugees and caseworkers, rather than the opposite poles of the controversial social representations of immigrants widely diffused in different societies. Those representations are highly dependent on the echo-chamber effect in the media, created by the discursive social positioning of the political leaders and governments, orienting their inclusive or exclusionary policies towards the immigrants. Results from de Rosa’s wide research program based on field and media studies on social representations [44,49,52,86,87,88,89,90] “show how the multiple denominations of migrants—also corresponding to different legal status (refugee, immigrants, stateless, newcomer, alien, undocumented, asylum-seeker) are often merged in the discourse ‘for’ and ‘by’ lay people in polarised representations of the immigrants as ‘unknown’, ‘foreign’ often associated in a one-sided manner with concepts such as ‘dangerous’, ‘extraneous’ and generally presented in the media as ‘invaders,’ even stigmatised as ‘criminal’ or potential ‘terrorists’—legitimising ‘fear’ and evoking the ‘need for barriers’ and protection of one’s own territory by the population of the host country. From the other side, however, they are viewed as ‘forced new home seekers’ and social victims” [44].

The mirror effect transformed into experiential empathy ensures that the dual representation engendered by the caseworkers does not generate an “othering” process of exclusion. Rather, it generates “otherness” inclusive identification. In this case, the “refugees” are not simply anonymous, un-named and de-personalised people or just numbers in the statistics of migratory flows; they are the “other” who reflects “me” as citizen of this planet as a “shared home”.

### 4.4. Implications for Practice and Future Research

#### 4.4.1. Implications for Practice

According to our data, mobilizing refugees’ social representations as a tool for reclaiming the emancipation of their common sense as valid knowledge [79] could improve social interventions with refugees. This perspective, centered on refugees’ knowledge and experience, would have the power to trigger a change in the relationship between caseworkers and their social work users, which would then become less paternalistic and lead to the emergence of “service user–expert” knowledge [91]. The “service user–expert” is the social service user who has the resilience, confidence, assets, skills, and knowledge to play an active role in managing their own life.

Our data also suggest the need for a better practical and explicit definition of the exact meaning of the integration of refugees in the host society. National immigration policies, political agendas, social expectations and the discourse of multiculturalism compel caseworkers in Canada to act in the ways imposed by their status as representatives of the host country. At times, the integration imperatives that underpin this migration regime [92] engender beliefs among the public [93] that integration means cultural assimilation [94]. Although multiculturalism is well embedded in Canadian identity [95,96], the discourse of integration through acculturation is self-perpetuating and continues to shape actions towards these newcomers [97]. From this perspective, it seems important to make explicit the ideological and political assumptions associated with the assertion that immigrants must integrate.

Additionally, our data suggest the need for better professional training for caseworkers with a migration background, especially in the field of mental health, and the possibility for caseworkers to debrief after an intervention with refugees in order to mitigate the negative consequences of the mirror effect on their well-being.

#### 4.4.2. Future Research

Future developments in this study may overcome some of the limitations of the present investigation by

(a) Increasing the number of research participants to make comparisons between sub-groups of caseworkers, depending on their higher or lower affinity in terms of the geo-cultural area of origin of their own migratory experience, spoken language, previous work experience and age compared with their target group of refugees;
(b) Increasing the number of professionals to be interviewed, depending on their professional involvement with the refugees’ relocation, also including native Canadian caseworkers with no personal migratory experience to be compared with the research participants with a migration background interviewed in this study;
(c) Comparing institutional policies towards migrants and refugees in different Western countries with different exposure to migratory flows, for example, by comparing the Canadian policies for the integration of immigrants and refugees with those adopted in the USA under presidents of different political orientations or with those adopted at a supranational level by the European Commission and by different EU member states in Europe exposed to different routes of migratory flows through the Mediterranean Sea or the Balkan routes (45,52,53,87–91).
(d) Conducting a longitudinal study to explore:
  d.1. the adjustment processes of the research participant’s experience, depending on their level of expertise in working with immigrants and refugees;
  d.2. the potential changes in the resilient or stressful work process, depending on potential shift from more inclusive towards more exclusionary policies adopted by governments of different political orientations both in Canada and in the specific provinces hosting the immigrants–refugees and ensuring working conditions and training support to the caseworkers, considering that previous studies of authors such as Barrington and Shakespeare-Finch [98], Robert et al. [99] or UNHCR [100] have identified government legislation as one of the greatest challenges affecting the mental health of caseworkers.

## 5. Conclusions

In 2015, Canada experienced several significant changes, such as a political shift towards a liberal policy on immigration, the resettlement of 25,000 Syrian refugees and the renaissance of a grassroots movement that redefined humanitarian action among their local communities. Moreover, local humanitarian organizations, much like international ones, tend to promote and maintain the idea of being the only means of rescue and assistance to refugees by perpetuating the image of refugees as vulnerable people who need to be cared for under the scrutiny of the media.

As with any forced displacement, resettlement and social assistance to these newcomers require logistical readiness from community-based organizations and from caseworkers, the professionals in the field. Being the main actors of the resettlement work, caseworkers are also the closest helping professionals to the refugees. This proximity can be professional but also social and cultural. Hence, this professional collaboration raises a panoply of images of the other, which are drawn from our social imagination and our social sharing of knowledge accumulated over our lifetimes. While developing and providing services, programs and activities for the integration of refugees, caseworkers with migration paths confront their images, their ideas, and their representations of refugees anchored in their past and in their professional practices and interactions.

Through the narratives of the participants, we have identified and highlighted a dual social representation of refugees: vulnerable refugees who suffer their fate, supported by specialized organizations that guide them through their migration trajectories, but at the same time, refugees as people who can demonstrate their resilience and find their ways to settle successfully in the host society.

The social positioning of the caseworkers as representatives of the host society generates a power relationship with the refugees, a relationship supported by the hegemonic representation of the refugees’ vulnerability. However, the migratory experience of the caseworkers is reflected in the refugees’ journey and generates the mirror effect, the resonance of the self in the other. This resonance diminishes the asymmetric nature of the relationship with the refugees. Refugees are therefore also represented as resilient and strong, aspects of their identity that are less recognized in social intervention. The mobilization of the refugees’ social representations in the intervention would facilitate the emancipation of their knowledge validated by their lived experience and thus their affirmation as autonomous persons able to succeed in their settlement in the host society.

## Data Availability

The data used in this study are available from the corresponding author upon request deemed reasonable by the authors.
